# Do protein crystals nucleate within dense liquid clusters?

**DOI:** 10.1107/S2053230X15008997

**Published:** 2015-06-27

**Authors:** Dominique Maes, Maria A. Vorontsova, Marco A. C. Potenza, Tiziano Sanvito, Mike Sleutel, Marzio Giglio, Peter G. Vekilov

**Affiliations:** a Structural Biology Brussels (SBB), Vrije Universiteit Brussel, 1050 Brussels, Belgium; b Department of Chemical and Biomolecular Engineering, University of Houston, Houston, TX 77204, USA; c Dipartimento de Fisica, Universita di Milano, 20133 Milano, Italy; d Department of Chemistry, University of Houston, Houston, TX 77204, USA

**Keywords:** nucleation, two-step mechanism, protein-rich clusters, crystallization

## Abstract

Protein-dense liquid clusters are regions of high protein concentration that have been observed in solutions of several proteins. The typical cluster size varies from several tens to several hundreds of nanometres and their volume fraction remains below 10^−3^ of the solution. According to the two-step mechanism of nucleation, the protein-rich clusters serve as locations for and precursors to the nucleation of protein crystals. While the two-step mechanism explained several unusual features of protein crystal nucleation kinetics, a direct observation of its validity for protein crystals has been lacking. Here, two independent observations of crystal nucleation with the proteins lysozyme and glucose isomerase are discussed. Firstly, the evolutions of the protein-rich clusters and nucleating crystals were characterized simultaneously by dynamic light scattering (DLS) and confocal depolarized dynamic light scattering (cDDLS), respectively. It is demonstrated that protein crystals appear following a significant delay after cluster formation. The cDDLS correlation functions follow a Gaussian decay, indicative of nondiffusive motion. A possible explanation is that the crystals are contained inside large clusters and are driven by the elasticity of the cluster surface. Secondly, depolarized oblique illumination dark-field microscopy reveals the evolution from liquid clusters without crystals to newly nucleated crystals contained in the clusters to grown crystals freely diffusing in the solution. Collectively, the observations indicate that the protein-rich clusters in lysozyme and glucose isomerase solutions are locations for crystal nucleation.

## Abstract 2

The evolution of protein-rich clusters and nucleating crystals were characterized by dynamic light scattering (DLS), confocal depolarized dynamic light scattering (cDDLS) and depolarized oblique illumination dark-field microscopy. Newly nucleated crystals within protein-rich clusters were detected directly. These observations indicate that the protein-rich clusters are locations for crystal nucleation.

## 1. Introduction   

Protein crystallization remains a challenge for structural biology and other areas relying on protein crystals (Chayen *et al.*, 2010 ▸; Bergfors, 2009 ▸; McPherson, 2009 ▸). In many instances, the problems can be traced to the unpredictability of crystal nucleation. Nucleation is the first step of crystallization, which determines the availability of crystals and several crucial properties of the crystal population: polymorph selection, number of crystals, crystal sizes and size distribution, and others. Hence, fundamental understanding of protein crystal nucleation has been an active area of investigation (Akella *et al.*, 2014 ▸; Arnold *et al.*, 1999 ▸; Ataka & Asai, 1990 ▸; Chayen, 2005 ▸; Chayen *et al.*, 2006 ▸; D’Arcy *et al.*, 2003 ▸; Dey *et al.*, 2010 ▸; Feher & Kam, 1985 ▸; Galkin & Vekilov, 1999 ▸, 2000*a* ▸,*b* ▸, 2001 ▸; Haas & Drenth, 2000 ▸; Lutsko *et al.*, 2010 ▸; Malkin & McPherson, 1994 ▸; Nanev & Penkova, 2001 ▸; Nicolis & Maes, 2012 ▸; Pan *et al.*, 2005 ▸; Pechkova & Nicolini, 2002 ▸; Penkova *et al.*, 2006 ▸; Reguera & Rubí, 2003 ▸; Sear, 2009 ▸; Selimović *et al.*, 2009 ▸; Shiryayev & Gunton, 2004 ▸; Sleutel *et al.*, 2014 ▸; Talanquer, 2005 ▸; ten Wolde & Frenkel, 1997 ▸; Tsekova *et al.*, 2002 ▸; Vekilov, 2004 ▸, 2005 ▸, 2010*a* ▸; Yau & Vekilov, 2001 ▸).

One of the significant recent findings in this pursuit has been the formulation of the two-step mechanism of nucleation (Galkin & Vekilov, 2000*b* ▸; Vekilov, 2004 ▸, 2010*a* ▸; Vekilov & Vorontsova, 2014 ▸; Chung *et al.*, 2010 ▸; Sauter *et al.*, 2015 ▸; Sleutel & Van Driessche, 2014 ▸). According to this mechanism, the nucleation of crystals is preceded by the formation of clusters of mesoscopic size composed of dense protein liquid (Gliko *et al.*, 2005*a* ▸, 2007 ▸; Pan *et al.*, 2007 ▸, 2010 ▸; Vekilov *et al.*, 2008 ▸). Crystal nuclei then form inside the clusters (Galkin & Vekilov, 2000*b* ▸; Garetz *et al.*, 2002 ▸; Vekilov, 2004 ▸). Protein-rich clusters that may be the nucleation precursors have been observed in solutions of several proteins: lysozyme, haemoglobin A and S, lumazine synthase, insulin and others. The size of the clusters typically varies from several tens to several hundreds of nanometres and their total volume fraction remains less than 10^−3^. It was shown that these clusters are present even in the homogeneous region of protein phase diagrams (Gliko *et al.*, 2005*a* ▸, 2007 ▸; Pan *et al.*, 2007 ▸, 2010 ▸; Li *et al.*, 2012 ▸) and their extended lifetimes indicate that they are not concentration fluctuations.

Direct imaging of crystal nuclei forming within dense liquid clusters has been provided for two types of systems: colloids, which are larger and move more slowly than most molecules (Savage & Dinsmore, 2009 ▸), and an ingeniously chosen organic system (Harano *et al.*, 2012 ▸). The evidence for the action of this two-step mechanism in the formation of nuclei of protein crystals (Vekilov, 2010*a* ▸; Sauter *et al.*, 2015 ▸), sickle-cell anaemia fibres (Galkin *et al.*, 2007 ▸) and amyloid fibrils (Lomakin *et al.*, 1996 ▸; Krishnan & Lindquist, 2005 ▸) has mostly been indirect: the two-step mechanism was put forth to explain unusual nonmonotonic dependencies of the protein crystal nucleation rate on supersaturation and a tenfold order-of-magnitude discrepancy between the nucleation rates predicted by the classical nucleation theory assuming one-step crystal nucleation and the actual data (Vekilov, 2004 ▸, 2010*a* ▸).

Nucleation of protein crystals and other ordered solids (*e.g.* sickle haemoglobin fibres) in stable dense protein liquid has been observed numerous times (Galkin *et al.*, 2002 ▸; Vivarès *et al.*, 2005 ▸). Direct observation of protein crystal nucleation inside the metastable clusters is challenging owing to a protein cluster size which typically is below the optical resolution limit. To tackle this challenge, in this work we monitored the initial progress of crystallization using two novel techniques: confocal depolarized dynamic light scattering (cDDLS) and depolarized oblique illumination dark-field microscopy (DOIDM). Monitoring of supersaturated protein solutions by depolarized optics allows the direct detection of crystals shortly after their formation. We demonstrate that the clusters are liquid in nature and that crystals always nucleate after clusters. We show that the motions of the nucleated crystals are not diffusive. We directly detect newly nucleated crystals within protein-rich clusters. We employed lysozyme for the cDDLS studies and glucose isomerase for the DOIDM tests.

## 2. Materials and methods   

### 2.1. The protein solutions   

Two stock solutions of hen egg-white lysozyme (Affymetrix, USA) were prepared: at a concentration of 138 mg ml^−1^ in 20 m*M* HEPES (*N*-2-hydroxyethylpiperazine-*N*′-2-ethanesulfonic acid) buffer pH 7.8 and at a concentration of 157 mg ml^−1^ in 50 m*M* sodium acetate buffer pH 4.5. Glucose isomerase from *Streptomyces rubiginosus* (Hampton Research, USA) was dialyzed against 100 m*M* HEPES buffer pH 7.0 containing 200 m*M* MgCl_2_. The concentrations of both proteins were determined by UV absorbance at 280 nm using extinction coefficients of 2.64 ml mg^−1^ cm^−1^ for lysozyme and 1.042 ml mg^−1^ cm^−1^ for glucose isomerase. All stock solutions were filtered through 0.22 µm syringe filters after preparation. For the crystallization experiments, supersaturation was achieved by adding sodium chloride and ammonium sulfate to the lysozyme and glucose isomerase solutions, respectively. All experiments were carried out at 22°C.

### 2.2. Confocal depolarized dynamic light scattering (cDDLS) in combination with dynamic light scattering (DLS)   

If a collection of birefringent scatterers is illuminated by a vertically polarized electric field *E*_V_, the scattered field can be described by two components, *E*_VV_ and *E*_VH_. The polarized *E*_VV_ is significantly more intense than the depolarized *E*_VH_ owing to the small birefringent power of the scatterers. When the scattered field is superimposed with the transmitted field, the scattered signal is heterodyned by the transmitted field, acting as a local oscillator. Unfortunately, since the scattered field is in quadrature with the transmitted field, this interference does not affect the intensity of the forward beam. To overcome this problem, traditionally a quarter-wave plate was inserted before the analyzer, so that only the scattered beam is phase-shifted by 90°, leading to a destructive interference affecting the measured intensity. We chose a different arrangement. We expanded the scattering amplitudes for birefringent scatterers, which promoted the second-order term to a quadrature of phase with the transmitted beam. The time fluctuations of both terms are synchronous. Therefore, no need arises for a quarter-wave plate: the amplitude of the second term is sufficiently large. We found that a confocal geometry, as described in Potenza *et al.* (2010 ▸, 2012 ▸), was the breakthrough: a strong second-order term ultimately requires a strong scattering efficiency, which leads to multiple scattering, the greatest limitation of the DDLS technique in the past. The cDDLS intensity scales with the anisotropy of the polarizability of the crystals, the number/size of crystals in the scattering volume and the relative refractive index of the crystals and the solution. Importantly, if all scatterers in the monitored volume are isotropic, no fluctuations are expected from the light collected in the forward direction.

**Figure 1:**
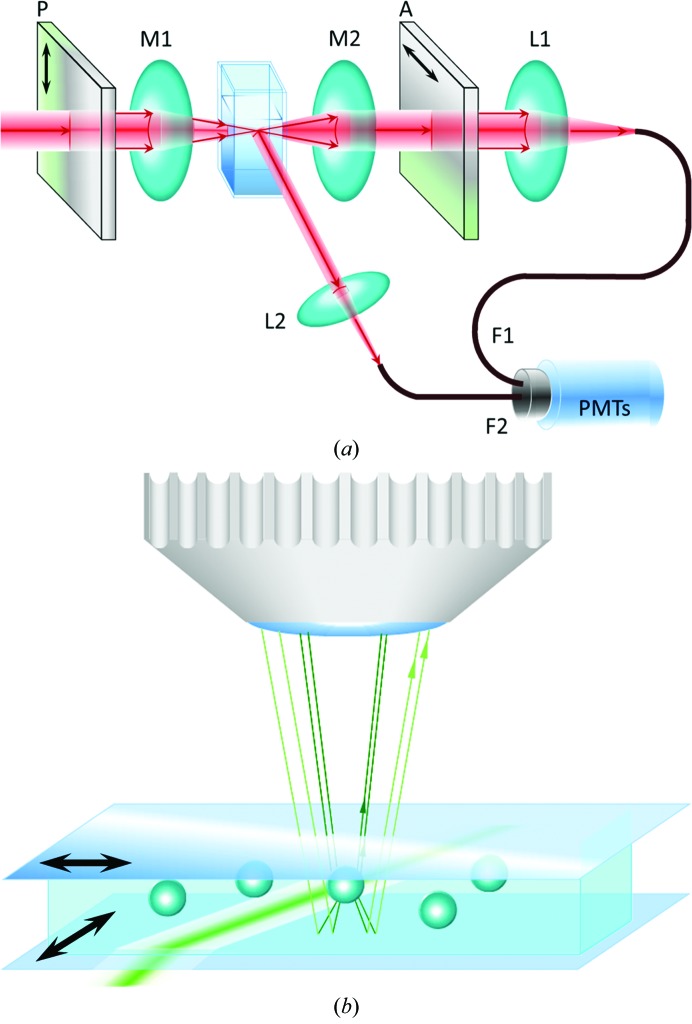
Schematic drawings of the experimental devices. (*a*) The experimental setup for combined dynamic light scattering (DLS) and confocal depolarized dynamic light scattering (cDDLS). A laser beam is polarized by the polarizer P and focused by lens M1 into the sample cell. Light scattered at 90° is introduced by lens L2 to an optical fibre F2 and transmitted to a photomultiplier (PMT). The transmitted light passing through an analyzer A at complete extinction with P is collected by lens L1 in a confocal scheme and sent by optical fibre F1 to a second photomultiplier. Both PMTs are connected to correlators. (*b*) Depolarized oblique illumination dark-field microscopy (DOIDM) setup. A laser beam illuminates a ∼100 µm wide path in a rectangular sample cell. The light scattered vertically is collected by an objective lens. A polarizer and analyzer (indicated by black arrows) are placed at the optical entrance and exit of the sample cell, respectively.

The experimental setup is shown in Fig. 1 ▸(*a*). A spatially filtered, collimated He–Ne laser beam (λ = 632.8 nm, 30 mW) passes through a Glan–Thompson polarizer (P) with vertical transmission. The beam is focused on the experimental cell volume (four optical windows, 2 mm optical path) *via* a microscope objective lens (Nachet 20×, numerical aperture 0.3). The DLS signal is collected at 90° by a lens (L2) and transmitted *via* a monomode optical fibre connected to a photomultiplier tube (PMT). For the cDDLS measurements the transmitted beam is sent to a collection optics identical to a confocal scheme where light is only collected from the volume illuminated by the focused beam. The forward scattered and the transmitted light are sent through an analyzer (A) at almost complete extinction, so that the whole depolarized signal can be superimposed on a small fraction of the transmitted beam projected in the horizontal direction. A second PMT collects the forward propagating light through a monomode optical fibre. The DLS and cDDLS signals from the respective photomultipliers are analyzed with digital multi-tau correlators, resulting in the DLS intensity and the cDDLS intensity autocorrelation functions. Details of the method are given in Potenza *et al.* (2010 ▸, 2012 ▸).

The cDDLS and DLS intensity autocorrelation functions *g*_2_ were acquired for 60 s. As in numerous previous investigations, the DLS correlation functions revealed the presence of two scatterers with distinct diffusion times: τ_1_ and τ_2_ (Gliko *et al.*, 2005*a* ▸, 2007 ▸; Pan *et al.*, 2007 ▸, 2010 ▸; Li *et al.*, 2011 ▸, 2012 ▸; Sleutel & Van Driessche, 2014 ▸; Vekilov & Vorontsova, 2014 ▸). In analogy to previous work, τ_1_ was assigned to the protein monomers, while τ_2_ was assigned to clusters. To determine τ_1_ and τ_2_ the DLS data were analyzed as described in Li *et al.* (2011 ▸) using the fitting function
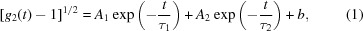
where *A*_1_ and *A*_2_ are their respective relative amplitudes; *b* is a baseline correction.

The cluster radius was determined from τ_2_ using the Stokes–Einstein relation; for details, see Pan *et al.* (2007 ▸) and Li *et al.* (2011 ▸). The viscosity of the solutions needed to evaluate the cluster radius was determined independently as described in Pan *et al.* (2007 ▸), Li *et al.* (2011 ▸) and Sleutel *et al.* (2012 ▸) by monitoring the diffusion of polystyrene spherical particles with diameter 600 nm.

The cDDLS data of interest (the interval between 0.001 and 2 s) were first smoothed by convolution with a normalized rect function. Two functions were fitted to the data: a single exponential function 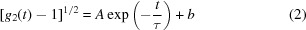
and a Gaussian function
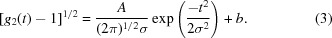



To evaluate the sensitivity of the method, we followed the discussion in Potenza *et al.* (2012 ▸). We calculated the scattering field matrix of a lysozyme crystal with an effective radius of 300 nm. We used the Amsterdam discrete-dipole approximation code, which is particularly suitable for this kind of numerical computation (Yurkin & Hoekstra, 2011 ▸). We obtained that the component of the adimensional field amplitude contributing to the cDDLS signal is of the order of 4 × 10^−5^. We compare this value with that obtained for a monodisperse suspension of MFA spheres 95 nm in diameter, which present a crystallinity of approximately 30%, as used for method validation in Potenza *et al.* (2010 ▸, 2012 ▸). In that earlier case, the same component of the adimensional field is less than 10^−6^. Still, particle size could be accurately determined with only *N* = 10 particles simultaneously present in the scattering volume. Note that owing to the heterodyne conditions of cDDLS, the random superposition of the field scales as *N* ^1/2^ (Potenza *et al.*, 2012 ▸). Thus, the resulting value for the field amplitude is smaller than 3 × 10^−6^, which is one order of magnitude less than we expect for a lysozyme crystal. These estimates indicate that the cDDLS method is sensitive to single lysozyme crystals.

### 2.3. Depolarized oblique illumination dark-field microscopy (DOIDM)   

A Nanosight LM10-HS microscope (Nanosight Ltd) equipped with a green laser (wavelength 532 nm) was employed to monitor individual clusters in the tested solutions (Fig. 1 ▸ *b*). The raw data of this method are movies of point-spread functions of clusters undergoing Brownian motion. To detect crystals, we modified the commercial setup by adding a polarizer at the optical entrance to the sample cell and an analyzer at the optical exit before the objective lens.

## 3. Results and discussion   

### 3.1. The protein-rich clusters   

**Figure 2:**
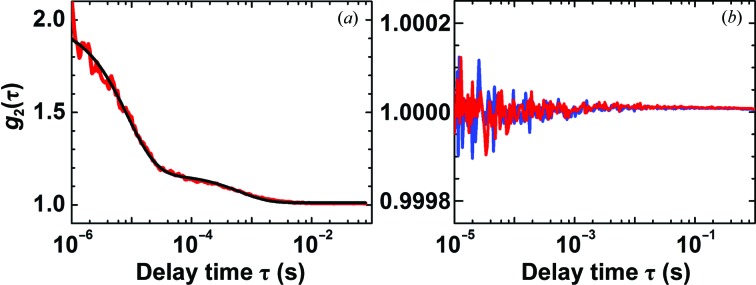
(*a*) A DLS intensity correlation function (red) of a lysozyme solution at 138 mg ml^−1^ in 20 m*M* HEPES buffer pH 7.8 and the fitted function (black). (*b*) cDDLS intensity correlation functions of the same lysozyme solution (red) and water (blue).

We simultaneously measured DLS and cDDLS intensity correlation functions of a lysozyme solution in undersaturated conditions (138 mg ml^−1^, 20 m*M* HEPES pH 7.8) where no crystals can nucleate (Fig. 2 ▸). Analyses of the DLS correlation functions revealed the presence of lysozyme monomers and clusters with characteristic diffusion times τ_1_ = 0.012 ms and τ_2_ = 1 ms, respectively. Using the Stokes–Einstein relation and accounting for the viscosities of the buffer, which scales the monomer radius, and of the protein solution, which scales the cluster radius, the corresponding hydrodynamic radii of these two scatterers are 1.5 and 43 nm, respectively. The monomer radius is close to the value determined from the atomic structure of the lysozyme molecule (Wang *et al.*, 2007 ▸). The mean cluster radius is consistent with that previously found under similar conditions (Li *et al.*, 2012 ▸; Pan *et al.*, 2010 ▸). The cDDLS correlation data from the protein sample are identical to those of water, indicating that the protein-rich clusters are liquid or amorphous solid objects.

### 3.2. Crystal nucleation within protein-rich clusters detected with cDDLS   

**Figure 3:**
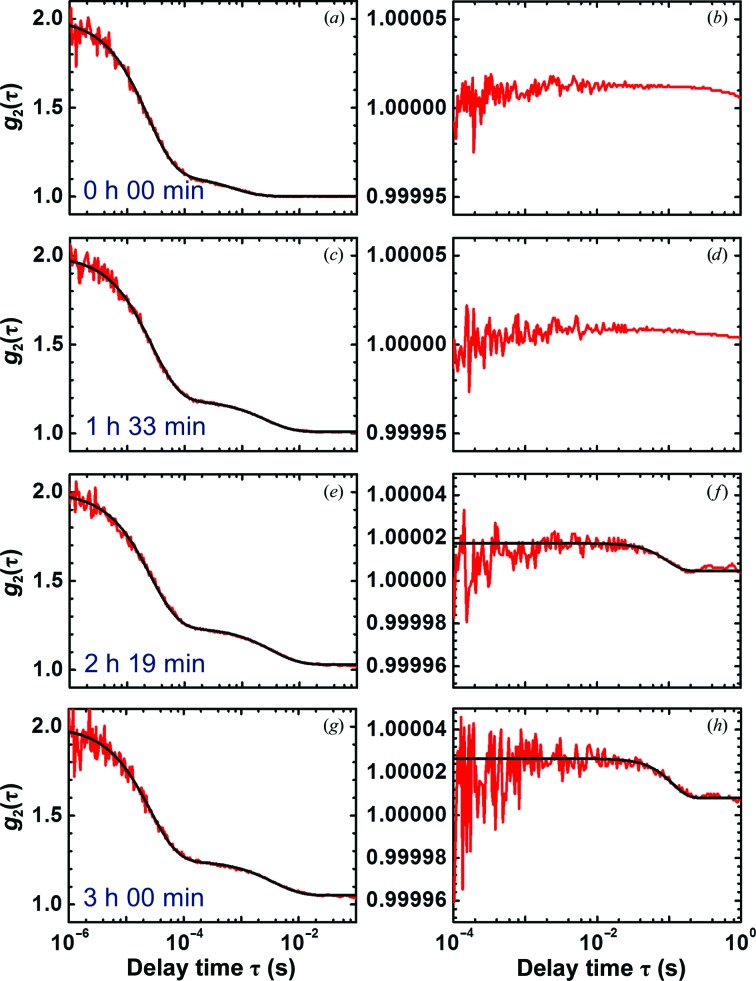
DLS (left column) and cDDLS (right column) intensity correlation functions recorded in parallel from the same lysozyme solution [50 mg ml^−1^ in 100 m*M* sodium acetate buffer pH 4.5 with 30 mg ml^−1^ (0.51 *M*) sodium chloride] at the times after onset of crystallization indicated in the plots. Before the experiment, all components of the mixture, water, buffer, salt and protein stock solution, were characterized separately. No cDDLS signal was detected. The fitted curves are indicated in black.

**Figure 4:**
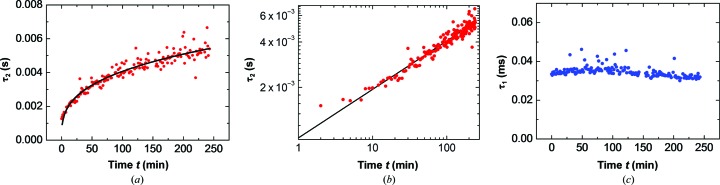
The evolution of the characteristic diffusion times of the clusters τ_2_ in (*a*) and (*b*) and monomers τ_1_ in (*c*) extracted from DLS correlation functions similar to those in Figs. 3 ▸(*a*), 3 ▸(*c*), 3 ▸(*e*) and 3 ▸(*g*) during the crystallization of lysozyme [50 mg ml^−1^ in 100 m*M* sodium acetate buffer pH 4.5 with 30 mg ml^−1^ (0.51 *M*) sodium chloride]. The time *t* is measured from the onset of the experiment. The black line in (*a*) and (*b*) depicts the *t* ^0.32^ dependence, which is close to the *t* ^0.33^ typical of Ostwald ripening.

To correlate the cluster characteristics with the nucleation of crystals, we simultaneously recorded cDDLS and DLS intensity correlation functions at regular time intervals after the preparation of a lysozyme solution supersaturated with respect to crystals (Fig. 3 ▸). The crystallization process was initiated by adding sodium chloride to a lysozyme solution in acetate buffer. Data collection was started immediately after mixing. The DLS correlation functions (several examples are displayed in Figs. 3 ▸ *a*, 3 ▸ *c*, 3 ▸ *e* and 3 ▸ *g*) revealed the presence of clusters from the onset of the experiment. The characteristic diffusion times of the clusters, determined from the entire sequence of correlation functions and displayed in Figs. 4 ▸(*a*) and 4 ▸(*b*), reveal that the cluster size increases in time *t* as *t* ^0.32^, while the characteristic diffusion time of the monomers τ_1_ in Fig. 4 ▸(*c*) is steady. The likely reason for the τ_2_ evolution is Ostwald ripening of the clusters, as observed for lysozyme clusters under similar conditions by Li *et al.* (2012 ▸). Ostwald ripening is driven by the minimization of the surface free energy of the cluster population (Ostwald, 1896 ▸; Lifshitz & Slyozov, 1961 ▸) so that smaller clusters disappear, while larger clusters grow on behalf of the released protein, resulting in growth of the mean cluster size and characteristic diffusion time τ_2_.

**Figure 5:**
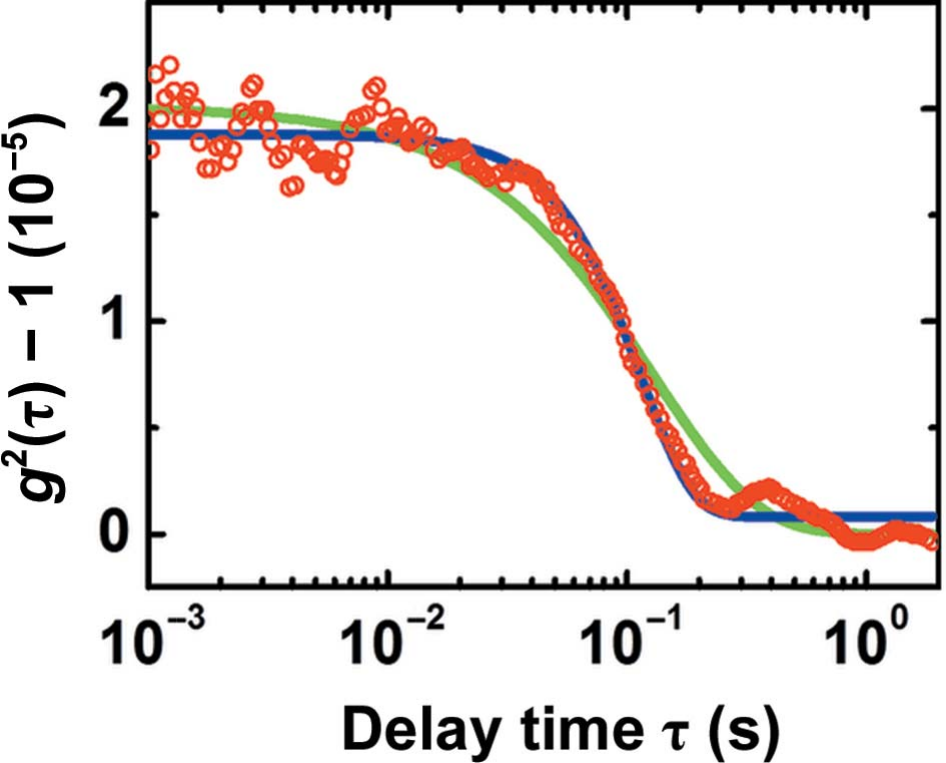
A typical cDDLS intensity correlation function (red symbols). Green line, best fit to data with an exponential decay function. Blue line, best fit to data with a Gaussian decay function.

The correlation functions of the depolarized cDDLS signal, sampled in Figs. 3 ▸(*b*), 3 ▸(*d*), 3 ▸(*f*) and 3 ▸(*h*), reveal the appearance of a shoulder with characteristic time ∼0.1 s after approximately 140 min (Figs. 3 ▸ *f* and 3 ▸ *h*). This shoulder indicates the presence of crystals in the cDDLS scattering volume. The cDDLS signal deviates from an exponential decay and is surprisingly well fitted with a Gaussian function (Fig. 5 ▸). The faster than exponential decay of the correlation function excludes the possibility that it reflects the polydispersity of the crystal population: polydispersity would have resulted in a stretched exponential decay. The non-exponential decay could be explained by assuming that the crystals participate in a nondiffusive motion. Three nondiffusive motions are possible: (i) the crystals are carried across the beam by convection owing to thermal gradients in the cell, (ii) the crystals are carried across the beam by sedimentation in the gravity field or (iii) the crystals are contained within dense liquid clusters that change shape.

To test scenarios (i) and (ii), we carefully analyzed the time sequence of the signal processed by the correlator. The characteristic time of decorrelation of the cDDLS signal in Figs. 3 ▸(*f*) and 3 ▸(*h*), 0.1 s, would require the crystal to pass through the cDDLS sampling width of 1 µm with a constant velocity of about 10 µm s^−1^. With respect to scenario (i) we note that at the very start of nucleation, where the concentration gradients are minor, convection could only be driven by thermal gradients and its velocity would be of the order of 0.1–1 µm s^−1^ (Lin *et al.*, 1995 ▸, 2001 ▸). This refutes scenario (i).

Concerning scenario (ii), in which the motion of the crystal is owing to sedimentation, the upper bound value of the difference in density between tetragonal lysozyme crystals (that form in the presence of sodium chloride) and the solution is ∼0.3 g cm^−3^ (Quillin & Matthews, 2000 ▸). From this, sedimentation velocities of the order of 10 µm s^−1^ would be attained by crystals of size 10 µm or larger. Such crystals would perturb the DLS signal and be detectable in it. Such perturbations are absent in the DLS correlation functions in Figs. 3(*e*) ▸ and 3(*g*) ▸.

Thus, scenario (iii), in which the crystals are contained within dense liquid clusters and are driven by the elastic response of the cluster surfaces to Brownian collisions with the solvent molecules, is a feasible explanation. As a first step in testing scenario (iii), below we explore the liquid nature of the clusters.

### 3.3. DOIDM characterization of clusters and crystal nucleation within clusters   

**Figure 6:**
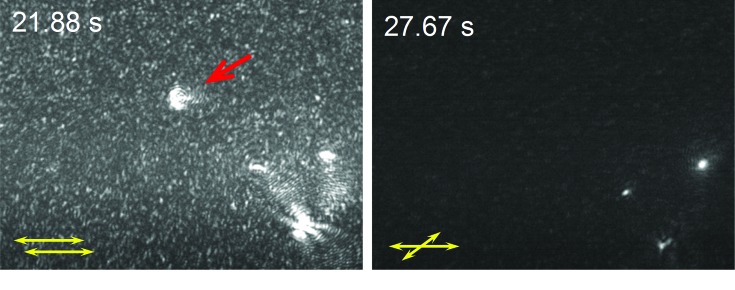
Depolarized oblique illumination dark-field microscopy (DOIDM) of a supersaturated glucose isomerase solution (90 mg ml^−1^ in 100 m*M* HEPES buffer pH 7.0 with 200 m*M* MgCl_2_ and 1 *M* ammonium sulfate). The two images were taken ∼40 min after the onset of the crystallization process in (*a*) parallel and (*b*) crossed polarizers. The time shown in the images is measured after the beginning of video recording. Judging from their changing shapes the intensity peaks in (*a*) are clusters. The cluster indicated by a red arrow in (*a*) is not seen in (*b*). The three intensity peaks in the bottom right corner of (*a*) are also seen in (*b*), suggesting that they represent small crystals within clusters. The time interval between the images is owing to the adjustment of the analyzer.

We tested the liquid nature of the protein-rich clusters suggested by previous indirect evidence (Gliko *et al.*, 2005*b* ▸, 2007 ▸; Pan *et al.*, 2007 ▸) with aged solutions of glucose isomerase, which hold protein-rich clusters of relatively large size (0.5–1.0 µm). Oblique illumination dark-field microscopy performed without a polarizer and analyzer exhibits unique intensity patterns that are readily distinguishable from those of solid protein aggregates, similar to those in Fig. 6 ▸(*a*). The patterns are dynamic and highly asymmetrical, with interference fringes spreading from the centre. The orientation of these fringes and their intensity vary with time. The minimum of the surface free energy for a liquid cluster corresponds to a spherical shape. However, Brownian collisions with the solvent molecules lead to deviations from this shape, resulting in the highly asymmetric dynamics of the intensity pattern. We conclude that the clusters are liquid and the surface free energy between the cluster and the solution is too low to stabilize a steady sphere. No determinations of the surface free energy of the protein-rich clusters have been performed. However, the free surface energy of dense liquid droplets in lysozyme solutions has been estimated as γ = 4 × 10^−5^ J m^−2^ (Vekilov, 2010*b* ▸). With this γ, the excess free energy of a protrusion of radius *r* = 100 nm can be estimated as Ωγ/*r* ≃ 10^−23^ J (Ω = 3 × 10^−26^ m^−3^ is the molecular volume). This is significantly less than the driving force for shape change, the thermal energy of the solvent molecules *k_B_T* ≃ 10^−21^ J (where *k*_B_ is the Boltzmann constant and *T* is the temperature). Assuming that the surface free energy of the protein-rich clusters in glucose isomerase solutions is similar to this value, shape dynamics in response to Brownian collisions with the solvent is feasible and the glucose isomerase clusters are liquid. If we transfer the conclusion of a liquid nature of the clusters to the clusters in lysozyme solutions, we provide the last missing link that attributes the non­diffusive cDDLS signal to crystals contained within clusters with liquid dynamic shape.

To study the nucleation of crystals, we added ammonium sulfate to glucose isomerase solution and loaded the solution into the DOIDM cell. If the polarizer and analyzer were in a parallel orientation we detected bright spots corresponding to individual clusters immediately after solution loading; a perpendicular arrangement of these two optical elements resulted in completely dark images. This indicates that only amorphous liquid clusters are present in the tested solutions. 40 min after the beginning of an experiment we see bright intensity spots with a crossed polarizer and analyzer, indicating the presence of objects that rotate the plane of polarization. Solution filtration prior to loading removed all stray crystals from the monitored volume; hence, we conclude that the only particles that may depolarize light are protein crystals. This signal indicates protein crystal nucleation. Further observations revealed three types of particles in the solution: (i) those visible only with a parallel polarizer and analyzer with a cluster-like intensity pattern, *i.e.* large protein clusters; (ii) those visible with both a parallel and a crossed polarizer and analyzer but with a steady shape; we conclude that these are large freely diffusing crystals; and (iii) particles that are visible with both a parallel and a crossed polarizer and analyzer but with a cluster-like pattern in parallel mode and a crystal-like pattern in crossed mode. We conclude that these are protein clusters with entrapped protein crystals inside.

In Fig. 6 ▸ we show two snapshots from a DOIDM movie, where we indicate the position of the analyzer and polarizer and the times corresponding to these frames. Fig. 6 ▸(*a*) is an example of the intensity patterns typically observed for large clusters in parallel polarizers. Fig. 6 ▸(*b*) shows three crystals in crossed polarizers seen as a steady bright spot. Comparing the locations of the crystals and three of the clusters, we conclude that these are most probably protein clusters with entrapped protein crystals inside.

## References

[bb1] Akella, S. V., Mowitz, A., Heymann, M. & Fraden, S. (2014). *Cryst. Growth Des.* **14**, 4487–4509.

[bb2] Arnold, S., Goddard, N. L. & Wotherspoon, N. (1999). *Rev. Sci. Instrum.* **70**, 1473–1477.

[bb3] Ataka, M. & Asai, M. (1990). *Biophys. J.* **58**, 807–811. 10.1016/S0006-3495(90)82425-5 PMC1281023 2207267

[bb4] Bergfors, T. (2009). *Protein Crystallization*, 2nd ed. La Jolla: International University Line.

[bb5] Chayen, N. E. (2005). *Prog. Biophys. Mol. Biol.* **88**, 329–337. 10.1016/j.pbiomolbio.2004.07.007 15652248

[bb6] Chayen, N. E., Helliwell, J. R. & Snell, E. H. (2010). *Macromolecular Crystallization and Crystal Perfection*, p. 24. Oxford University Press.

[bb7] Chayen, N. E., Saridakis, E. & Sear, R. P. (2006). *Proc. Natl Acad. Sci. USA*, **103**, 597–601. 10.1073/pnas.0504860102 PMC1334630 16407115

[bb8] Chung, S., Shin, S.-H., Bertozzi, C. R. & De Yoreo, J. J. (2010). *Proc. Natl Acad. Sci. USA*, **107**, 16536–16541. 10.1073/pnas.1008280107 PMC2944705 20823255

[bb9] D’Arcy, A., Mac Sweeney, A. & Haber, A. (2003). *Acta Cryst.* D**59**, 1343–1346. 10.1107/s0907444903009430 12832806

[bb10] Dey, A., Bomans, P. H. H., Müller, F. A., Will, J., Frederik, P. M., de With, G. & Sommerdijk, N. A. J. M. (2010). *Nature Mater.* **9**, 1010–1014. 10.1038/nmat2900 21076415

[bb11] Feher, G. & Kam, Z. (1985). *Methods Enzymol.* **114**, 77–112. 10.1016/0076-6879(85)14006-1 4079780

[bb12] Galkin, O., Chen, K., Nagel, R. L., Hirsch, R. E. & Vekilov, P. G. (2002). *Proc. Natl Acad. Sci. USA*, **99**, 8479–8483. 10.1073/pnas.122055299 PMC124280 12070342

[bb13] Galkin, O., Pan, W., Filobelo, L., Hirsch, R. E., Nagel, R. L. & Vekilov, P. G. (2007). *Biophys. J.* **93**, 902–913. 10.1529/biophysj.106.103705 PMC1913141 17449671

[bb14] Galkin, O. & Vekilov, P. G. (1999). *J. Phys. Chem. B*, **103**, 10965–10971.

[bb15] Galkin, O. & Vekilov, P. G. (2000*a*). *J. Am. Chem. Soc.* **122**, 156–163.

[bb16] Galkin, O. & Vekilov, P. G. (2000*b*). *Proc. Natl Acad. Sci. USA*, **97**, 6277–6281. 10.1073/pnas.110000497 PMC18593 10823898

[bb17] Galkin, O. & Vekilov, P. G. (2001). *J. Cryst. Growth*, **232**, 63–76.

[bb18] Garetz, B., Matic, J. & Myerson, A. (2002). *Phys. Rev. Lett.* **89**, 175501. 10.1103/PhysRevLett.89.175501 12398680

[bb19] Gliko, O., Neumaier, N., Pan, W., Haase, I., Fischer, M., Bacher, A., Weinkauf, S. & Vekilov, P. G. (2005*a*). *J. Am. Chem. Soc.* **127**, 3433–3438. 10.1021/ja043218k 15755162

[bb20] Gliko, O., Neumaier, N., Pan, W., Haase, I., Fischer, M., Bacher, A., Weinkauf, S. & Vekilov, P. G. (2005*b*). *J. Cryst. Growth*, **275**, e1409–e1416. 10.1021/ja043218k 15755162

[bb21] Gliko, O., Pan, W., Katsonis, P., Neumaier, N., Galkin, O., Weinkauf, S. & Vekilov, P. G. (2007). *J. Phys. Chem. B*, **111**, 3106–3114. 10.1021/jp068827o 17388477

[bb22] Haas, C. & Drenth, J. (2000). *J. Phys. Chem. B*, **104**, 368–377.

[bb23] Harano, K., Homma, T., Niimi, Y., Koshino, M., Suenaga, K., Leibler, L. & Nakamura, E. (2012). *Nature Mater.* **11**, 877–881. 10.1038/nmat3408 22983432

[bb24] Krishnan, R. & Lindquist, S. L. (2005). *Nature (London)*, **435**, 765–772. 10.1038/nature03679 PMC1405905 15944694

[bb25] Li, Y., Lubchenko, V. & Vekilov, P. G. (2011). *Rev. Sci. Instrum.* **82**, 053106. 10.1063/1.3592581 21639491

[bb26] Li, Y., Lubchenko, V., Vorontsova, M. A., Filobelo, L. & Vekilov, P. G. (2012). *J. Phys. Chem. B*, **116**, 10657–10664. 10.1021/jp303316s 22889282

[bb27] Lifshitz, I. M. & Slyozov, V. V. (1961). *J. Phys. Chem. Solids*, **19**, 35–50.

[bb28] Lin, H., Petsev, D. N., Yau, S.-T., Thomas, B. R. & Vekilov, P. G. (2001). *Cryst. Growth Des.* **1**, 73–79.

[bb29] Lin, H., Rosenberger, F., Alexander, J. I. D. & Nadarajah, A. (1995). *J. Cryst. Growth*, **151**, 153–162.

[bb30] Lomakin, A., Chung, D. S., Benedek, G. B., Kirschner, D. A. & Teplow, D. B. (1996). *Proc. Natl Acad. Sci. USA*, **93**, 1125–1129. 10.1073/pnas.93.3.1125 PMC40042 8577726

[bb31] Lutsko, J. F., Basios, V., Nicolis, G., Kozak, J. J., Sleutel, M. & Maes, D. (2010). *J. Chem. Phys.* **132**, 035102. 10.1063/1.3294561 20095752

[bb32] Malkin, A. J. & McPherson, A. (1994). *Acta Cryst.* D**50**, 385–395. 10.1107/S0907444993013319 15299390

[bb33] McPherson, A. (2009). *Introduction to Macromolecular Crystallo­graphy*. Hoboken: Wiley–Blackwell.

[bb34] Nanev, C. N. & Penkova, A. (2001). *J. Cryst. Growth*, **232**, 285–293.

[bb35] Nicolis, G. & Maes, D. (2012). Editors. *Kinetics and Thermodynamics of Multistep Nucleation and Self-Assembly in Nanoscale Materials*. Hoboken: John Wiley & Sons.

[bb36] Ostwald, W. (1896). *Lehrbuch der Allgemeinen Chemie.* Leipzig: Engelmann.

[bb37] Pan, W., Galkin, O., Filobelo, L., Nagel, R. L. & Vekilov, P. G. (2007). *Biophys. J.* **92**, 267–277. 10.1529/biophysj.106.094854 PMC1697867 17040989

[bb38] Pan, W., Kolomeisky, A. B. & Vekilov, P. G. (2005). *J. Chem. Phys.* **122**, 174905. 10.1063/1.1887168 15910067

[bb39] Pan, W., Vekilov, P. G. & Lubchenko, V. (2010). *J. Phys. Chem. B*, **114**, 7620–7630. 10.1021/jp100617w 20423058

[bb40] Pechkova, E. & Nicolini, C. (2002). *J. Cell. Biochem.* **85**, 243–251. 10.1002/jcb.10123 11948680

[bb41] Penkova, A., Pan, W., Hodjaoglu, F. V. & Vekilov, P. G. (2006). *Ann. N. Y. Acad. Sci.* **1077**, 214–231. 10.1196/annals.1362.048 17124126

[bb42] Potenza, M. A. C., Sanvito, T., Alaimo, M. D., Degiorgio, V. & Giglio, M. (2010). *Eur. Phys. J. E*, **31**, 69–72. 10.1140/epje/i2010-10550-2 20087622

[bb43] Potenza, M., Sanvito, T., Degiorgio, V. & Giglio, M. (2012). *Kinetics and Thermodynamics of Multistep Nucleation and Self-Assembly in Nanoscale Materials*, edited by G. Nicolis & D. Maes, pp. 61–78. Hoboken: John Wiley & Sons.

[bb44] Quillin, M. L. & Matthews, B. W. (2000). *Acta Cryst.* D**56**, 791–794. 10.1107/s090744490000679x 10930825

[bb45] Reguera, D. & Rubí, J. M. (2003). *J. Chem. Phys.* **119**, 9888.

[bb46] Sauter, A., Roosen-Runge, F., Zhang, F., Lotze, G., Jacobs, R. M. J. & Schreiber, F. (2015). *J. Am. Chem. Soc.* **137**, 1485–1491 10.1021/ja510533x 25569484

[bb47] Savage, J. R. & Dinsmore, A. D. (2009). *Phys. Rev. Lett.* **102**, 198302. 10.1103/PhysRevLett.102.198302 19519003

[bb48] Sear, R. P. (2009). *J. Chem. Phys.* **131**, 074702. 10.1063/1.3205030 19708753

[bb49] Selimović, S., Jia, Y. & Fraden, S. (2009). *Cryst. Growth Des.* **9**, 1806–1810. 10.1021/cg800990k PMC2714170 20161207

[bb50] Shiryayev, A. & Gunton, J. D. (2004). *J. Chem. Phys.* **120**, 8318–8326. 10.1063/1.1695321 15267753

[bb51] Sleutel, M., Lutsko, J., Van Driessche, A. E. S., Durán-Olivencia, M. A. & Maes, D. (2014). *Nature Commun.* **5**, 5598. 10.1038/ncomms6598 PMC4268696 25465441

[bb52] Sleutel, M. & Van Driessche, A. E. S. (2014). *Proc. Natl Acad. Sci. USA*, **111**, E546–E553. 10.1073/pnas.1309320111 PMC3918807 24449867

[bb53] Sleutel, M., Van Driessche, A. E. S., Pan, W., Reichel, E. K., Maes, D. & Vekilov, P. G. (2012). *J. Phys. Chem. Lett.* **3**, 1258–1263. 10.1021/jz300459n 26286768

[bb54] Talanquer, V. (2005). *J. Chem. Phys.* **122**, 084704. 10.1063/1.1851508 15836074

[bb55] Tsekova, D., Popova, S. & Nanev, C. (2002). *Acta Cryst.* D**58**, 1588–1592. 10.1107/s0907444902014452 12351867

[bb56] Vekilov, P. G. (2004). *Cryst. Growth Des.* **4**, 671–685.

[bb57] Vekilov, P. G. (2005). *J. Cryst. Growth*, **275**, 65–76.

[bb58] Vekilov, P. G. (2010*a*). *Cryst. Growth Des.* **10**, 5007–5019. 10.1021/cg1011633 PMC2995260 21132117

[bb59] Vekilov, P. G. (2010*b*). *Soft Matter*, **6**, 5254–5272.

[bb60] Vekilov, P. G., Pan, W., Gliko, O., Katsonis, P. & Galkin, O. (2008). *Aspects of Physical Biology: Biological Water, Protein Solutions, Transport and Replication*, edited by G. Franzese & M. Rubi, pp. 65–95. Heidelberg: Springer.

[bb61] Vekilov, P. G. & Vorontsova, M. A. (2014). *Acta Cryst.* F**70**, 271–282. 10.1107/S2053230X14002386 PMC3944685 24598910

[bb62] Vivarès, D., Kaler, E. W. & Lenhoff, A. M. (2005). *Acta Cryst.* D**61**, 819–825. 10.1107/S090744490402949X 15930647

[bb63] Wang, J., Dauter, M., Alkire, R., Joachimiak, A. & Dauter, Z. (2007). *Acta Cryst.* D**63**, 1254–1268. 10.1107/S0907444907054224 18084073

[bb64] Wolde, P. R. ten & Frenkel, D. (1997). *Science*, **277**, 1975–1978. 10.1126/science.277.5334.1975 9302288

[bb65] Yau, S.-T. & Vekilov, P. G. (2001). *J. Am. Chem. Soc.* **123**, 1080–1089. 10.1021/ja003039c 11456661

[bb66] Yurkin, M. A. & Hoekstra, A. G. (2011). *J. Quant. Spectrosc. Radiat. Transfer*, **112**, 2234–2247.

